# The Acoustic Structure and Information Content of Female Koala Vocal Signals

**DOI:** 10.1371/journal.pone.0138670

**Published:** 2015-10-14

**Authors:** Benjamin D. Charlton

**Affiliations:** School of Biology and Environmental Science, University College Dublin (UCD), Belfield, Dublin, Ireland; University of York, UNITED KINGDOM

## Abstract

Determining the information content of animal vocalisations can give valuable insights into the potential functions of vocal signals. The source-filter theory of vocal production allows researchers to examine the information content of mammal vocalisations by linking variation in acoustic features with variation in relevant physical characteristics of the caller. Here I used a source-filter theory approach to classify female koala vocalisations into different call-types, and determine which acoustic features have the potential to convey important information about the caller to other conspecifics. A two-step cluster analysis classified female calls into bellows, snarls and tonal rejection calls. Additional results revealed that female koala vocalisations differed in their potential to provide information about a given caller’s phenotype that may be of importance to receivers. Female snarls did not contain reliable acoustic cues to the caller’s identity and age. In contrast, female bellows and tonal rejection calls were individually distinctive, and the tonal rejection calls of older female koalas had consistently lower mean, minimum and maximum fundamental frequency. In addition, female bellows were significantly shorter in duration and had higher fundamental frequency, formant frequencies, and formant frequency spacing than male bellows. These results indicate that female koala vocalisations have the potential to signal the caller’s identity, age and sex. I go on to discuss the anatomical basis for these findings, and consider the possible functional relevance of signalling this type of information in the koala’s natural habitat.

## Introduction

Determining the information content of a given species’ vocal signals is of prime importance to researchers because it might indicate different communicative functions [1, 2]. The source-filter theory of vocal production allows researchers to probe the information content of mammalian vocal signals because it explicitly links specific acoustic features of calls to their production mechanisms. According to the source-filter theory, mammal vocal signals are generated by the conversion of airflow from the lungs to acoustic energy by the larynx, the source, which is subsequently filtered by the vocal tract. The source signal determines the fundamental frequency (F0) of the vocalisation and the supra-laryngeal vocal tract acts as a spectral filter, selectively amplifying certain frequencies of the source signal before it radiates out through the mouth and/or nostrils. The broadband frequency peaks that result from this filtering process are termed vocal tract resonances or ‘formants’ [3]. The source-filter theory, therefore, provides a framework which allows researchers to identify acoustic characteristics that have the potential to provide receivers with direct information about a given caller’s physiological and/or morphological attributes [4, 5], because any acoustic variation can be directly linked to variation in relevant aspects of the caller’s phenotype (such as the vocaliser’s size, age, sex or hormonal state).

For example, using a source-filter theory approach several studies on humans and non-human mammals have confirmed that F0 [6–9] and formants [10–17] are individually distinctive components of vocalisations. In addition, formants provide accurate information to receivers on the caller’s body size in a range of mammals [18–25]. This relationship exists because lower and more closely spaced formants indicate longer vocal tracts, and the length of the vocal tract, being constrained by the bones of the skull, is typically correlated to body size [26]. Relative differences in formant spacing (or dispersion) in species-specific calls may also allow conspecific receivers to gauge the caller’s maturity [19] and sex in size dimorphic species [25, 27]. In addition, because laryngeal development and the visco-elastic properties of vocal fold tissue are affected by the sex hormones [28–30] the F0 of a vocalisation may also provide information about a caller’s sex [31], and various age-related changes in vocal fold mass, stiffness and length could provide cues to a caller’s age [3, 25]. It is also important to note that irregular or chaotic vocal fold vibration patterns can produce various forms of nonlinear phenomena (NLP) in the spectral acoustic structure of vocal signals [32–34]. These include additional spectral components called *subharmonics* that suddenly appear at integer fractional values of an identifiable F0, (e.g., F0/2, F0/3 etc.), abrupt discontinuous changes in F0 called *frequency jumps*, episodes of non-random noise termed *deterministic chaos*, and the occurrence of two simultaneous but independent fundamental frequencies, termed *biphonation*. NLP can reflect vocal fold pathology, but is also theorized to have potential adaptive significance in animal vocal communication systems, ranging from increasing the individual distinctiveness of vocalisations [35, 36] to grabbing the attention of receivers and preventing them from habituating to repetitively produced calls [37–40].

The koala (*Phascolarctos cinereus*) is one of the most vocal of all Australian marsupials. Initial studies of male and female koala vocal behaviour described the vocal repertoire and documented the different behavioural contexts of call production [41, 42]. More recent studies have investigated how male koala bellows mediate spatial distribution in free-ranging animals during the breeding season [43], and examined the information content and function of these calls [15, 24, 44–47]. In contrast, detailed information about the acoustic structure and function of female koala vocal signals is lacking. Nonetheless, female koalas are also known to produce bellow vocalisations during the reproductive period [42], particularly when they are in oestrous [48], and deliver loud squawks, screams, squeaks, wails and snarls when they reject male copulation attempts [41, 42] or rebuff the advances of oestrous females that occasionally mount other communally housed individuals [49]. Due to the context of delivery, female squawks, screams, squeaks, wails and snarls are often collectively referred to as “rejection” calls [41, 48]. Female koalas are also reported to produce low amplitude grunts in response to mild disturbances (such as being handled or disturbed by another animal climbing over them) [42] and oestrous barks [48], although these could be the same call-type judging by the published descriptions of these vocalisations.

The koalas’ arboreal and mostly solitary lifestyle means that effective communication is likely to be crucial for coordinating reproductive activities. Interestingly, although this species’ mating system is polygamous [41, 50], data from wild populations indicates that male koalas only sire on average a maximum of two offspring in a given breeding season [51]. In line with these observations, the male koala’s quite small testes relative to body size [52] also suggest a monoandrous system in which females typically mate with a single male during an oestrous cycle. Thus, due to the relatively low reproductive potential of male koalas when compared to other polygynous male mammals (such as red deer and elephant seals [53, 54]), it could prove adaptive for them to exercise a degree of choice over mating partners if it is possible to do so. Indeed, the fecundity of female koalas decreases with age after sexual maturity is reached [55], and work on the reproductive success of paired dyads in captivity suggests that male koalas are also less likely to copulate with females that are 5–7 years older than them [56]. The study by Bercovitch and colleagues [56] also revealed that males and females which had previously copulated with one another were significantly more likely to successfully copulate during subsequent breeding introductions. As a consequence, information about the identity and age of female koalas may have functional significance for males during the breeding season. For example, acoustic cues to identity and age in female calls might allow males to approach females that they have previously bred with and are, therefore, more likely to be receptive to their copulation attempts, and/or younger females that are more fecund. In addition, if consistent acoustic differences between male and female bellows exist, male koalas could distinguish between the bellows of opposite sex conspecifics that represent possible mating partners versus same-sex conspecifics, and preferentially locate and approach the former during the breeding season.

The goal of the current study was twofold: the first aim was to objectively classify vocalisations into different call-types based solely on their acoustic structure and provide a detailed acoustic description of female koala vocal signals using a source-filter theory approach. The second aim was to determine the information content of female koala vocalisations: specifically, I investigated whether the acoustic features of female koala vocal signals have the potential to convey information about the identity and age of female callers, and also compared the acoustic features of female bellows with male bellows from a previously published dataset [15] to establish whether acoustic cues to the caller’s sex exist within this call-type.

## Materials and Methods

### Ethical statement

This work follows the Association for the study of Animal Behaviour/Animal Behaviour Society guidelines for the use of animals in research, and was approved by the University of Queensland Animal Ethics Committee (approval number SUSSEX/SAFS/436/12). The owner of LPKS issued permission for the research on captive koalas to be conducted by BDC. The research did not affect the housing, diet or management of the animals.

### Study site and population

Recordings were obtained during the 2011 breeding season (September–December) from 23 adult female koalas aged 2–14 years (mean = 5.88) at Lone Pine Koala Sanctuary (LPKS), Brisbane, Queensland, Australia. The male koala bellows used for the analysis of sex differences in bellows were recorded at LPKS during the 2010 breeding season from 20 males aged 3–15 years [15]. All the koalas in the study were individually recognisable and of known age. This allowed the identity of a vocalising animal to be noted at the same time recordings were captured, so that the age and sex of individuals could be retrospectively obtained from LPKS husbandry records.

### Recordings

**Fig 1:**
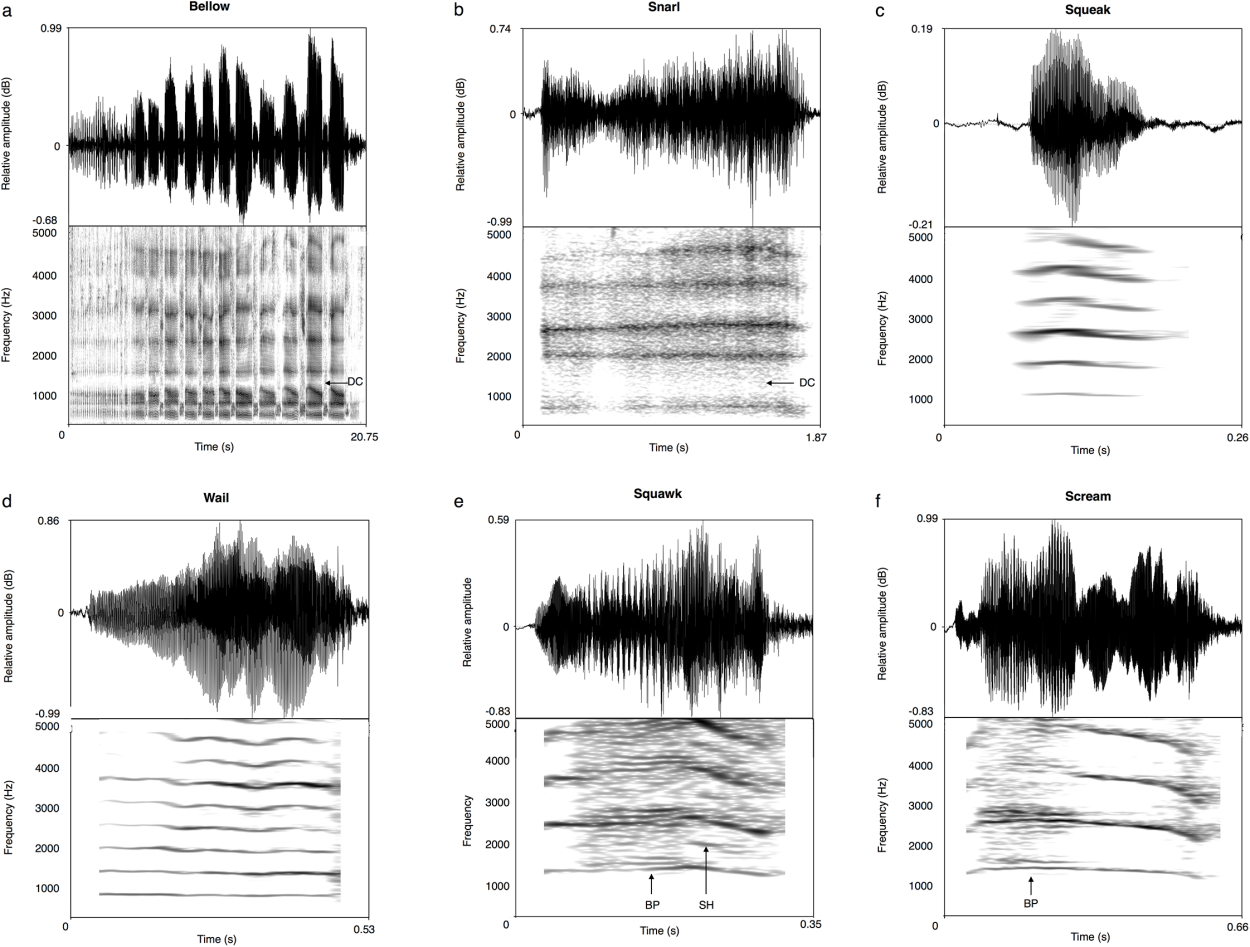
Waveforms and spectrograms of female koala vocalisations. Spectrogram settings: Fast Fourier Transform (FFT) method; window length = 0.03 s; time step = 0.002; frequency step = 20 Hz; Gaussian window shape; dynamic range = 40 dB. The two-step cluster analysis and grouped female tonal rejection calls squawks (b), squeaks (c), wails (d) and screams (e), together. Bellows (a) and snarls (f) were separately clustered as discrete call-types. NLP is signified as follows: DC = deterministic chaos, BP = biphonation, SH = subharmonics.

Koala vocalisations were recorded using a Sennheiser ME67 (Sennheiser Electronics, Wedemark, Germany) directional microphone attached to a Zoom H4N portable solid-state digital recorder (Tokyo, Japan; sampling rate 44.1 kHz, amplitude resolution 16 bits) at distances ranging from 5–20 metres. The recordings were then normalized to 100% peak amplitude, converted to mono, and saved as WAV files (44.1 kHz sampling rate and 16 bits amplitude resolution). The overall spectral structure of each call was initially investigated using narrow-band spectrograms (see Fig 1: FFT method, window length = 0.03 s, time steps = 250, frequency steps = 1000, Gaussian window shape, dynamic range = 40 dB) and recordings were split into bellows, snarls squawks, squeaks, wails and screams based on qualitative descriptions of these calls [42]. It was not possible to capture recordings of female grunts or oestrous barks.

### Acoustic Analyses

The acoustic analyses were performed on 355 female vocalisations using custom built programs in Praat 5.3.85 DSP package [57] that automatically extract and measure a range of acoustic measures (see Table 1). The outputs were checked against the corresponding spectrograms to ensure that Praat accurately tracked and measured all acoustic features. In addition, because female koala vocalisations exhibited clear NLP, visual inspection of narrow-band spectrograms allowed me to document whether subharmonics, deterministic chaos and biphonation were present in calls. Other types of NLP such as frequency jumps were not observed.

**Table 1:** Descriptive statistics for each of the acoustic measures. See text for definition of variables.

	Bellows (N = 115)				Tonal rejection calls (N = 212)				Snarls (N = 28)			
Acoustic measures	*M*	s.d.	Minimum	Maximum	*M*	s.d.	Minimum	Maximum	*M*	s.d.	Minimum	Maximum
Duration (s)	19.4	9.9	6.9	70.9	0.5	0.5	0.1	2.6	0.9	0.3	0.3	1.7
Mean F0 (Hz)	31.3	12.7	12.3	63.8	792.1	193.6	257.2	1184.4	0.0	0.0	0.0	0.0
Maximum F0 (Hz)	68.9	25.5	12.7	104.3	842.1	197.3	305.8	1276.3	0.0	0.0	0.0	0.0
Minimum F0 (Hz)	10.5	5.2	0.1	54.3	741.3	194.0	139.2	1123.2	0.0	0.0	0.0	0.0
F0 sumvar (Hz)	58.5	26.8	0.0	98.8	604.9	475.0	6.6	2421.2	0.0	0.0	0.0	0.0
Deterministic Chaos (% of calls)	100.0	0.0	100.0	100.0	7.5	0.0	0.0	100.0	100.0	0.0	100.0	100.0
Subharmonics (% of calls)	0.0	0.0	0.0	0.0	33.5	0.0	0.0	100.0	0.0	0.0	0.0	0.0
Biphonation (% of calls)	0.0	0.0	0.0	0.0	46.2	0.0	0.0	100.0	0.0	0.0	0.0	0.0
GO (Hz)	0.0	0.0	0.0	0.0	186.3	0.0	73.1	275.7	0.0	0.0	0.0	0.0
F1 (Hz)	259.9	23.5	221.0	323.0	0.0	0.0	0.0	0.0	824.2	212.6	587.0	1468.0
F2 (Hz)	511.8	29.7	450.0	596.0	0.0	0.0	0.0	0.0	2130.8	249.6	1583.0	2500.0
F3 (Hz)	743.0	42.1	644.0	858.0	0.0	0.0	0.0	0.0	3273.7	264.2	2739.0	3783.0
F4 (Hz)	1313.7	69.9	1084.0	1458.0	0.0	0.0	0.0	0.0	4413.5	266.7	3899.0	4870.0
F5 (Hz)	1874.1	71.0	1701.0	2059.0	0.0	0.0	0.0	0.0	5463.4	242.7	5049.0	5991.0
F6 (Hz)	2634.9	91.4	2421.0	2846.0	0.0	0.0	0.0	0.0	6243.1	168.4	5895.0	6557.0
ΔF (Hz)	423.5	9.9	397.4	454.1	0.0	0.0	0.0	0.0	1205.1	42.3	1114.0	1291.0

#### a) Analysis of bellows

Koala bellows typically have an introductory phase consisting of abrupt amplitude onsets and offsets produced on exhalation, followed by a continuous series of inhalations and shorter exhalations [15, 24, 42]. The exhalation sections of bellows are characterised by deterministic chaos with no apparent F0 or harmonic structure, whereas the later inhalation sections have a very clear F0 and formant structure [15, 24]. Accordingly, and following previous studies [15, 24], only the later inhalation sections of bellows with a clear F0 and stable formants were considered for the analysis.

For female bellows the F0 of the inhalation sections was extracted using a search range of 10–100 Hz and a time step of 0.01. Time-varying numerical representations of the F0 contour were then checked for any incorrect values before the mean, minimum, and maximum F0 values (mean F0, minimum F0 and maximum F0, respectively), and amount of F0 modulation per second (F0 sumvar) were measured. Extracted F0 contours were also played back (as a pulse train) for subjective comparison with the original recording [15]. The duration of bellows was measured directly from the waveform and any NLP documented. To measure the frequency values of the first six formants (F1-F6) I used Linear Predictive Coding (LPC; ‘To Formants (Burg)’ command in Praat) and the following analysis parameters: time step, 0.01 s; window analysis, 0.03 s; maximum formant value, 3000 Hz; maximum number of formants, 6; and pre-emphasis, 50 Hz. Prior to the formant analysis visual inspection of spectrograms allowed me to confirm that the lower six formants of each bellow fell below the maximum formant value setting (of 3000 Hz). The formant values were then used to estimate the formant spacing (ΔF) during each bellow using the linear regression method [19]. Exactly the same approach was used to measure the acoustic features of male koala bellows except the maximum formant value was set at 2300 Hz (for more details see [15]).

#### b) Analysis of female rejection calls

For tonal female rejection calls with an observable fundamental frequency (squawks, screams, squeaks and wails) the F0 contour was extracted using the To Pitch (cc) command in Praat and the following parameters were measured: mean F0, minimum F0, maximum F0, and F0 sumvar. The time step in the analysis was 0.01 seconds and the minimum and maximum values for tracking the F0 contour were set at 180–2500 Hz. In cases where a harmonic or a sub-harmonic were tracked instead of F0 (octave jumps), numerical representations of the F0 contour were manually adjusted using the ‘Edit’ window in Praat, before the resulting F0 contour was played back for comparison with the original recording. If subharmonics were observed the ratio of subhamonics to F0 was noted (F0/2, F0/3 etc). In addition, where biphonation was detected, the mean frequency of the second independent periodic signal was measured and termed G0. Biphonation can be seen as two distinct and independently varying frequency contours or as sidebands adjacent to harmonics that are associated with cyclic amplitude fluctuations in the waveform [32, 34]. Because the biphonation observed in female koala calls manifested itself as clear amplitude modulation in the waveform, it was measured using a pulse train analysis in Praat that automatically counts the number of pulses occurring per second.

Formants were not observed in tonal female rejection calls due to their relatively high F0 and the concomitant decreased harmonic density that fails to adequately sample these spectral components [9, 58]. In addition, source-related features were not extracted from non-tonal female rejection calls (i.e. snarls) because they consist of broadband frequency noise without an observable F0. Distinct energy bands were observed within the broadband frequency noise of snarls, however, that are likely to represent formants. To measure these spectral peaks (hereafter termed F1-F6) I used Linear Predictive Coding (LPC; ‘To Formants (Burg)’ command in Praat) and the following analysis settings: time step, 0.01 s; window analysis, 0.03 s; maximum formant value, 7000 Hz; maximum number of formants, 6; and pre-emphasis, 50 Hz. The mean frequency values of the spectral peaks (F1-F6) were then used to estimate the their overall spacing (ΔF) during each snarl using the linear regression method of Reby and McComb [19]. Call duration was measured directly from the waveform.

### Statistical analyses

All statistical analyses were conducted using IBM SPSS Statistics version 20, significance levels were set at 0.05, and two-tailed probability values are quoted. A two-step cluster analysis was first of all performed to confirm the provisional classification of female calls into six different call-types (bellow, snarl, squawk, squeak, wail and scream) based on visual inspections of spectrograms and previous descriptions of call characteristics [42]. Two-step clustering was necessary due to the combination of binary (dichotomous) and continuous acoustic measures. A two-step cluster analysis partitions the data into a predefined number of relatively homogenous groupings in order to minimise variability within clusters and maximise variability between clusters. For the analyses, I used the log-likelihood distance measure and set the number of clusters from 2–6 to compare silhouette information and determine the best solution (with the highest silhouette measure). A maximum of six clusters were used because it corresponded to the provisional subjective classification of the calls.

To evaluate individual differences in the acoustic structure of female koala vocalisations I then performed separate discriminant functions analyses (DFAs) on the different call-types (determined by the cluster analyses), with subject identity as the group identifier and the acoustic measures as discriminant variables. For all DFAs the percentage correct classification expected due to chance was automatically calculated by SPSS according to the number of vocalisations each subject had in the analysis (by ticking “compute from group sizes” for prior probabilities). Wilk’s lambda is then used to assess the level of correct classification of calls to different female koalas (where “1” indicates no differentiation between individuals and “0” indicates perfect or complete differentiation) and the statistical significance obtained using the chi-square distribution [59]. Both the reclassification and the more conservative leave-one-out cross-validation procedure were applied for all the DFAs. The statistical significance of correct classification using the leave-one-out cross-validation procedure was obtained using a Binomial test in which the observed percentage of correct classification to different individuals is compared to that expected by chance. To determine whether the acoustic structure of female rejection calls varied according to the age or sex of the caller I ran two separate multivariate general linear models (MANOVAs), in which age or sex were entered as the independent variable and the mean acoustic measures for each subject were entered as dependant variables. Univariate tests conducted at the same time to examine the affect of age and sex on individual acoustic characteristics. The directions of any effects of age on individual acoustic features were determined using the slope of standardized beta coefficients.

## Results

### Classification of female calls

Visual examination of female koala calls using narrow-band spectrograms resulted in six provisional call-types: bellows, snarls, squeaks, squawks, wails and screams (Fig 1). The two-step cluster analysis solution with the highest silhouette measure (of 8.0) was obtained using three clusters: the 1^st^ cluster consisted entirely of bellows, the 2^nd^ cluster comprised all the snarls, and the 3^rd^ cluster contained the tonal rejection calls (squeaks, wails, squawks and screams). The grouping of the tonal female koala rejection calls into one cluster confirms that these calls lie on a continuum of highly graded vocalisations [42]. Based on the results of the cluster analysis, female vocalisations are subsequently referred to as bellows, snarls, and tonal rejection calls.

### Acoustic characteristics of female calls

Descriptive statistics for all the source- and filter-related features of female koala vocalisations are given in Table 1. A description of the acoustic characteristics of female koala vocalisations now follows:

#### a) Bellows

**Fig 2:**
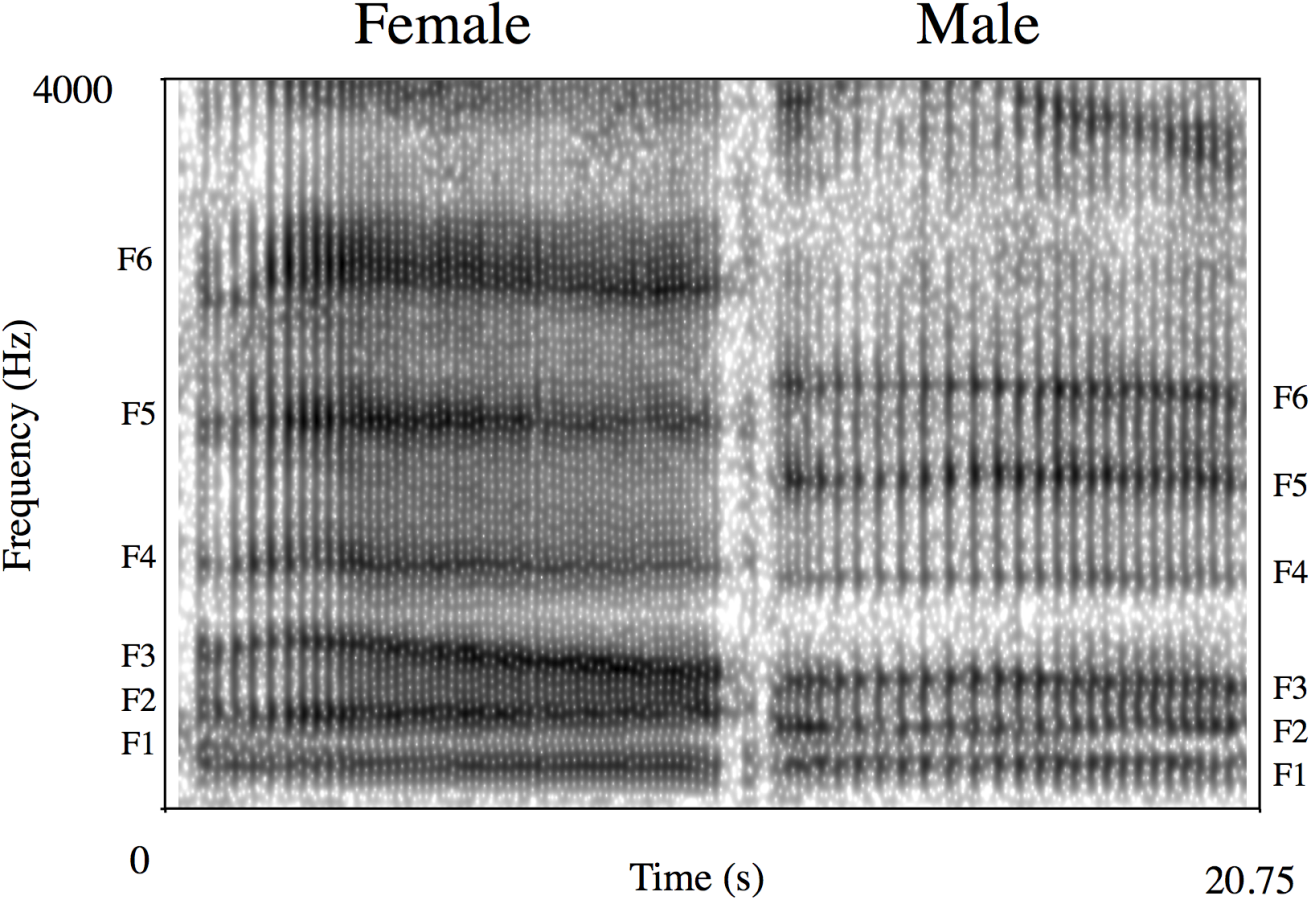
Comparison of female and male bellow inhalation sections. Spectrogram settings: Fast Fourier Transform (FFT) method; window length = 0.03 s; time step = 0.002; frequency step = 20 Hz; Gaussian window shape; dynamic range = 40 dB). The dark energy bands labelled (F1-F6) are the formant frequencies. Note that they are higher in female bellows.

Female bellows consisted of a series of inhalation and exhalation components and ranged in duration from 6.9 to 70.9 seconds (mean = 19.4 s) (Table 1), making them the longest duration of all the female koala’s documented vocalisations (Fig 1A). The exhalation sections of female bellows contained deterministic chaos/broadband noise and no clear harmonic structure, whereas inhalation sections had a very low F0 (mean = 31.3 Hz), making a pulse-train structure clearly visible in the spectrogram and waveform. In addition, there were clear spectral peaks in the inhalation and exhalation phases of female bellows that are likely to represent formants (Fig 2). The mean formant spacing was 423.5 Hz (Table 1). The only form of NLP observed in female bellows was deterministic chaos, and this was present in 100% of calls Table 1). The lack of other NLP indicates that the vibration pattern of the oscillating sound source is very stable. In most respects female koala bellows are structurally similar to male koala bellows [15, 24] (Fig 2). It is worth noting though, that the “staccato” introductory phase often seen at the beginning of male bellows was less commonly observed in female bellows.

#### b) Snarls

Snarls were characterised by broadband frequency noise with no observable harmonic structure and six clear spectral peaks below 7000 Hz that seem likely to represent formants (Fig 1B). The average spacing of the spectral peaks ranged from 1114.0–1291.0 Hz (mean = 1205.1 Hz) (Table 1). These calls also had the second longest mean call duration of 0.90 s (range = 0.3–1.7 s) and 100% (28/28) contained deterministic chaos (Table 1). No other forms of NLP were observed in snarls.

#### c) Tonal rejection calls

Tonal rejection calls comprise the squeaks, squawks, wails and screams previously described by Smith [42] (Fig 1C–1F). The duration of tonal rejection calls varied from 0.1–2.6 s (mean = 0.5 s) and the mean F0 features were 790.3 Hz, 739.7 Hz, and 840.1 Hz for mean, minimum and maximum F0 respectively (Table 1). F0 also ranged widely across the dataset, varying from a minimum value of 139.3 Hz to a maximum value of 1276.3 Hz (Table 1). The F0 modulation rate (F0 sumvar) of around 604.2 Hz per second indicates that tonal rejection calls are also quite strongly modulated. Formants were not observed in any tonal rejection calls but all three types of NLP were detected. Biphonation was found in 46.2% of calls (98/212): with a G0 (the second independent frequency) of around 186 Hz and ranging up to 275.7 Hz (Table 1). The presence of biphonation produced clear periodic amplitude modulation in the waveform as well as sidebands in the spectrogram (Fig 1E and 1F). Subharmonics were also noted in 33.5% (71/212) of calls (Table 1), 51 occasions at F0/2 and 18 occasions at F0/3. Deterministic chaos was only found in 16 out of 212 of tonal rejection calls (7.5%) (Table 1).

### The information content of female koala calls

#### a) Individual differences in acoustic structure

For the analysis of individual differences I only considered females that contributed at least four calls for each call-type. The dataset consisted of 115 bellows from 12 females (5–18 each), 212 tonal rejection calls from 14 females (4–57 each), and 28 snarls from six females (4–9 each). The DFA correctly classified 85.2% of bellows to the 12 individuals. This level of correct classification was significantly higher than that expected by chance (Wilk’s Lambda = 0.004, *χ* ^*2*^ = 573.64, df = 110, *P* < 0.001). In addition, 66.5% of tonal rejection calls were correctly classified to the 14 individuals, and 78.6% of snarls to the five individuals. Again, both of these classification rates are significantly above chance levels (tonal rejection calls: Wilk’s Lambda = 0.049, *χ* ^*2*^ = 603.48, df = 117, *P* < 0.001; snarls: Wilk’s Lambda = 0.044, *χ* ^*2*^ = 65.50, df = 28, *P* < 0.001). When the more conservative leave-one-out, cross validation was applied classification levels dropped to 63.5% for bellows (chance classification rate = 8% (1/12), Binomial Test: *P* < 0.001), 56.6% for tonal rejection calls (chance classification rate = 7% (1/14), Binomial Test: *P* < 0.001), and 0.0% for snarls (chance classification rate = 20% (1/5), Binomial Test: *P* = 0.193), suggesting that female snarls are the least individually distinctive of the three call-types. The univariate analyses, however, showed that all the acoustic features of tonal rejection calls and snarls that were measured differed significantly between individuals (all *P* < 0.05) (Table 2). Call duration and the filter-related features of bellows (F1-F6, ΔF) also differed significantly according to the caller’s identity (all *P* < 0.05), whereas source-related features (mean F0, minimum F0, maximum F0, and F0 sumvar) did not (Table 2). The structure matrices generated by the multivariate DFAs showed that the upper formants (F4-6) and ΔF were most important for classifying bellows and snarls to individuals (Table 3), whereas F0 features and call duration contributed the most to the individuality of tonal rejection calls (see Table 3 for more information on the variance explained by each of the first three discriminant factors and the loading of each acoustic measure on these factors).

**Table 2:** Tests of equality of group means between individuals for the acoustic measures derived from each of the three call-types. “-”= cannot be computed because this variable is constant.

Call type	Acoustic measures	Wilks' lambda	*F*	DF1	DF2	*P*
Bellows	Duration	0.656	4.905	11	103	< 0.001
	Mean F0	0.912	0.904	11	103	0.539
	Maximum F0	0.909	0.94	11	103	0.506
	Minimum F0	0.889	1.17	11	103	0.317
	F0 sumvar	0.912	0.898	11	103	0.545
	Deterministic Chaos	-	-	-	-	-
	F1	0.661	4.799	11	103	< 0.001
	F2	0.687	4.263	11	103	< 0.001
	F3	0.415	13.177	11	103	< 0.001
	F4	0.582	6.714	11	103	< 0.001
	F5	0.343	17.915	11	103	< 0.001
	F6	0.343	17.903	11	103	< 0.001
	ΔF	0.468	10.649	11	103	< 0.001
Snarls	Duration	0.539	4.92	4	23	0.005
	Deterministic Chaos	-	-	-	-	-
	F1	0.555	4.607	4	23	0.007
	F2	0.544	4.827	4	23	0.006
	F3	0.663	2.923	4	23	0.043
	F4	0.424	7.827	4	23	< 0.001
	F5	0.537	4.962	4	23	0.005
	F6	0.627	3.428	4	23	0.024
	ΔF	0.45	7.033	4	23	0.001
Tonal rejection calls	Duration	0.368	26.155	13	198	< 0.001
	Mean F0	0.352	27.982	13	198	< 0.001
	Maximum F0	0.392	23.652	13	198	< 0.001
	Minimum F0	0.367	26.216	13	198	< 0.001
	F0 sumvar	0.751	5.047	13	198	< 0.001
	Deterministic Chaos	0.803	3.74	13	198	< 0.001
	Subharmonics	0.698	6.59	13	198	< 0.001
	Biphonation	0.724	5.81	13	198	< 0.001
	G0	0.703	6.424	13	198	< 0.001

**Table 3:** DFA structure matrix. The pooled within-groups correlations between discriminating variables and the first three standardized canonical discriminant functions are shown. Discriminating variables are ordered by absolute size of correlation within function. Correlation coefficients > 0.4 are shown.

	Bellows			Tonal rejection calls			Snarls		
Acoustic measures	Discriminant functions			Discriminant functions			Discriminant functions		
	1	2	3	1	2	3	1	2	3
Duration (s)			-0.64	-0.61	0.68				0.78
Mean F0 (Hz)			0.63	0.66	0.65				
Maximum F0 (Hz)			0.57	0.59	0.62				
Minimum F0 (Hz)			0.64	0.66	0.52				
F0 sumvar (Hz)									
Deterministic Chaos (% of calls)						0.43			
Subharmonics (% of calls)						0.58			
Biphonation (% of calls)									
GO (Hz)									
F1 (Hz)								0.55	
F2 (Hz)								0.71	
F3 (Hz)								0.52	-0.67
F4 (Hz)							0.65		-0.46
F5 (Hz)	-0.41						0.50		
F6 (Hz)		0.68					0.42		0.45
ΔF (Hz)		0.47					0.59		
Eigenvalue	4.87	2.86	1.56	3.20	0.40	.55	3.16	1.46	1.12
% of Variance	43.5	25.5	13.9	61.1	20.7	7.7	54.6	25.2	19.4
Cumulative %	43.5	68.9	82.9	61.1	81.8	89.4	54.6	79.9	99.2

#### b) The effect of age on the acoustic characteristics of female calls

Age did not significantly affect the acoustic structure of female bellows (N = 12, Wilks’ Lambda = 0.223, *F*_1, 10_ = 1.553, *P* = 0.559), tonal rejection calls (N = 14, Wilks’ Lambda = 0.143, *F*_4, 9_ = 2.674, *P* = 0.178), or snarls (N = 5, Wilks’ Lambda = 0.148, *F*_1, 4_ = 8.621, *P* = 0.244). However, univariate tests did reveal that bellows from older females had higher minimum F0 (*P* = 0.033), and that older female koalas produced tonal rejection calls with lower mean F0, minimum F0 and maximum F0 (all P < 0.05) (Table 4). The presence of deterministic chaos also tended to be higher in the tonal rejection calls of younger individuals (P = 0.063) (Table 4).

**Table 4:** Age versus acoustic features of female koala vocalisations. Significant correlations are highlighted in bold. “*b”* = standardized beta coefficient.

	Bellows			Tonal rejection calls			Snarls		
Acoustic measures	*b*	*F*_1, 10_	*P*	*b*	*F*_4, 9_	*P*	*b*	*F*_1, 3_	*P*
Duration (s)	-1.04	2.43	0.15	-0.01	0.12	0.73	0.05	1.35	0.33
Mean F0 (Hz)	0.94	3.83	0.08	-37.23	9.54	**0.01**			
Maximum F0 (Hz)	-0.61	0.28	0.61	-34.11	8.71	**0.01**			
Minimum F0 (Hz)	0.48	6.08	**0.03**	-41.10	8.88	**0.01**			
F0 sumvar (Hz)	-1.09	0.90	0.36	28.72	0.39	0.55			
Deterministic Chaos (% of calls)				-0.01	4.21	0.06			
Subharmonics (% of calls)				0.03	0.67	0.43			
Biphonation (% of calls)				-0.05	2.81	0.12			
GO (Hz)				-12.29	3.91	0.07			
F1 (Hz)	1.49	0.85	0.38				-42.74	5.70	0.10
F2 (Hz)	2.65	1.24	0.29				-28.73	0.89	0.42
F3 (Hz)	0.70	0.03	0.87				-12.09	0.26	0.64
F4 (Hz)	9.12	2.55	0.14				-51.07	1.73	0.28
F5 (Hz)	3.43	0.15	0.71				-48.44	3.63	0.15
F6 (Hz)	9.34	1.03	0.33				-14.49	0.60	0.50
ΔF (Hz)	1.47	2.28	0.16				-7.96	1.73	0.28

#### c) Sex differences in the acoustic structure of bellows

For this analysis I compared the mean acoustic values of 276 bellows from 20 male koalas (mean 13.8 per male) and 115 bellows from 12 female koalas (mean = 9.6 per female). The multivariate tests revealed a highly significant effect of sex on the acoustic structure of bellows (Wilks’ Lambda = 0.087 *F*_10, 21_ = 21.914, *P* < 0.001). The univariate tests showed that female koala bellows were significantly shorter in duration than male bellows (*P* < 0.01) (Table 5). In addition, all source and filter-related features of female bellows except minimum F0 (*P* = 0.165) were higher in frequency than they were in male bellows (Table 5).

**Table 5:** Comparison of means between acoustic features of male and female koala bellows. Significant differences are highlighted in bold.

	Mean ± SD			
Acoustic feature	Females (*N* = 12)	Males (*N* = 20)	*F*_10, 21_	*P*
Duration (s)	18.97 ± 5.63	38.50 ± 12.23	26.89	**< 0.01**
Mean F0 (Hz)	31.97 ± 4.28	27.07 ± 5.77	6.48	**0.02**
Maximum F0 (Hz)	70.38 ± 8.88	61.45 ± 12.18	4.87	**0.04**
Minimum F0 (Hz)	10.48 ± 1.89	9.80 ± 0.76	2.03	0.17
F1 (Hz)	259.51 ± 12.82	216.94 ± 17.85	51.87	**< 0.01**
F2 (Hz)	511.06 ± 19.23	416.68 ± 28.92	100.43	**< 0.01**
F3 (Hz)	745.83 ± 31.20	660.79 ± 55.26	23.68	**< 0.01**
F4 (Hz)	1322.82 ± 48.68	1155.96 ± 134.51	16.94	**< 0.01**
F5 (Hz)	1886.32 ± 68.49	1618.40 ± 105.97	60.96	**< 0.01**
F6 (Hz)	2633.09 ± 73.51	2131.99 ± 143.23	125.78	**< 0.01**
ΔF (Hz)	424.64 ± 8.21	355.81 ± 23.12	97.78	**< 0.01**

## Discussion

The results of this study provide a quantitative description of the acoustic structure of female koala vocalisations. The acoustic data presented describe one temporal feature, 12 source- and filter-related acoustic measures, and three types of nonlinear phenomena present in female koala calls (Table 1). The current study is therefore more extensive than previous work on this species, which did not use a source-filter theory approach to link different frequency components of female calls to their production mechanisms [42]. The results also revealed that female koala vocal signals contain information about a given caller’s phenotype that may be of importance to receivers. Specifically, I found that both source-and filter-related acoustic features of female koala vocal signals were individually distinctive, and F0 related features of female calls were negatively correlated to the caller’s age. In addition, male and female koala bellows differed significantly in acoustic structure, and therefore have the potential to signal information about the caller’s sex.

### Classification of female koala vocalisations

Female koala vocal signals have previously been described and subjectively classified into different call-types. In this study, a cluster analysis was used to objectively classify female vocalisations into three call-types based solely on their acoustic structure. The findings accord well with those of previous studies, with female koala bellows and snarls classified as discrete call-types, and the tonal female rejection calls (i.e. those with a clear F0) grouped together due to their overlapping acoustic features. Although female koala squeaks, wails, squawks and screams are clearly highly-graded vocalisations, it would be possible to further sub-divide tonal rejection calls into those with and without biphonation, as only squawks and screams displayed this type of NLP. Recordings of oestrous barks or grunts were not captured in the current study and could therefore not be described here. Based on the qualitative descriptions of these short duration low amplitude calls though, they may represent the same call-type produced by females at different times of the reproductive cycle (oestrus versus non-oestrus). Future studies should aim to determine whether or not this is the case.

### Acoustic characteristics of female koala vocalisations

The acoustic characteristics of female koala vocalisations can be directly related to their production mechanisms. Previous work on male koala vocal anatomy and bellow acoustics has shown that males use a previously undiscovered non-laryngeal sound source to produce the very low F0 that characterises bellow inhalation sections, termed the velar vocal folds [60]. The current study has revealed that female koala bellows also have a very low F0 of around 31 Hz. If vocal fold tissue is considered to behave like a simple string (e.g. [3, 61–64]) the F0 of vocal fold vibration can be approximated using the following equation:

$$F0=\frac{1}{2L}\sqrt{\frac{\sigma }{\rho }}$$ (1)

in which (L) is the vocal fold length in m, σ is the stress applied to the vocal folds in kPa and ρ is the tissue density (1.02 g/cm3 [62, 63]). In theory then, the lowest possible F0 for a given length fold can be estimated if we assume that no stress (tension) is applied to the vocal folds during sound production. Female koala vocal fold length is around 7.8 mm (unpublished data), which is, in principle, incompatible with the production of frequencies lower than 63.3 Hz if we apply Eq 1. Consequently, the most parsimonious explanation is that female koalas also have velar vocal folds that they use to produce the very low F0 observed in their bellows. In addition, because the F0 features of female bellows are higher in frequency than those of male bellows, the oscillating structures that produce these acoustic features are probably smaller in females. Future anatomical studies should confirm whether female koalas also possess velar vocal folds, and relate the dimensions of these structures to the F0 characteristics of female bellows described here.

The inhalation sections of female koala bellows also contain very low frequency spectral peaks that seem likely to represent formants, as they do in male bellows [24]. These spectral prominences are not harmonically related and show the same general formant frequency pattern of male bellows (Fig 2). Hence, they are extremely unlikely to be remnants of the harmonic structure (called pseudo-formants [65]). The formant spacing (ΔF) of female bellows was 423.5 Hz. If we use the following equation eVTL = c/2ΔF, in which eVTL is the estimated vocal tract length of the caller, c = the speed of sound in warm air (350 m/s) and ΔF is the formant spacing, an estimated vocal tract length of 41.3 cm is derived. This is clearly a much longer vocal tract than expected for an animal the size of a koala. The formant spacing of male bellows (of 353.7 Hz) also predicts an extremely long VTL of 49.5 cm [24], and is thought to be produced as resonances of the oral and nasal tract are simultaneously excited to provide more formants within a given frequency range. A combination of anatomical studies and vocal tract modelling are now required to investigate the production of the unusually low formants in male and female koala bellows.

Female snarls contained broadband frequency noise with no F0 or harmonic structure, which indicates aperiodic vocal fold vibration during call production. I suggest that the high motivational state associated with these calls, which are produced when females aggressively rebuff copulation attempts [42], results in high sub-glottal air pressures that force vocal fold vibration to desynchronise. The noisy broadband frequency sound source is, however, ideal for highlighting vocal tract resonances, which are clearly observed in these calls. Interestingly, the spectral peaks in snarls likely to represent vocal tract resonances have an average spacing of 1205.1 Hz, which corresponds to an eVTL of 14.5 cm. MRI and post-mortem data derived from dead specimens indicates that the male koala’s oral vocal tract length (from the opening of the glottis to the tip of the lips) is around 13.5 cm [24]; however, female koalas noticeably round their lips when they snarl [42], which would effectively lengthen the oral tract by an additional 1–2 cm. If we assume that the female koala also has an oral vocal tract length of between 12–13 cm (without vocal tract elongation via lip rounding), the eVTL derived from the spacing of the spectral peaks in snarls indicates that these are indeed vocal tract resonances (or formants), and that these calls are most likely to be produced purely using the vocal tract as a resonator, in contrast to bellows in which the extremely low formants seem more likely to be the product of simultaneous resonators (as discussed by [24]). Although the findings of the current study support the hypothesis that the spectral peaks in female snarls are indeed formants, it must be noted that to prove this beyond any doubt would require experiments that record female koalas vocalising in heliox (a mixture of helium and air) which leads to an upward shift in formants whilst leaving other acoustic features unchanged [66].

The F0 characteristics of tonal rejection calls are consistent with a laryngeal source. F0 ranged from a lowest minimum F0 value of 139.3 Hz to a highest maximum F0 value of 1276.3 Hz, which is compatible with the female koala’s vocal fold length of 7.9 mm (unpublished data). For example, female rhesus macaques have a vocal fold length of 7.8 mm and produce coo calls that range in F0 from 80–2000 Hz [62]. In addition, if we use the string equation to model vocal fold vibration a 7.9 mm fold should be able to oscillate periodically at frequencies down to 63.3 Hz. Periodic oscillation at 1300 Hz would require a stress of 430.3 kPa to be applied to the female koala’s vocal folds (solving Eq 1 for σ), which is certainly a realistic value when compared to documented stress-strain relationships in other mammals [62, 63, 67].

Subharmonics were detected in a third (33.3%) of all tonal rejection calls. This form of NLP is produced when one of the vocal folds oscillates at integer ratios of the other (e.g. 1:2, 1:3) resulting in additional frequency components appearing at integer fractional values of an identifiable F0. The relatively high prevalence of subharmonics in tonal rejection calls may increase the direct auditory impact of these calls [40], but also suggests that vocal fold vibration in the female koala is prone to instability, perhaps due to tension asymmetry across the vocal folds during call production causing partial desynchronisation of the folds [68]. A high proportion of tonal female koala rejection calls also contained biphonation (two independent frequencies). In calls with biphonation, the second independent frequency G0 had a mean value of 184.9 Hz and ranged down to a minimum of 73.1 Hz for one individual. Since it is theoretically possible for the female koala’s vocal folds to produce this frequency range (according to the string equation), biphonation in female koala rejection calls may occur as a consequence of the vocal folds decoupling completely during call production, allowing them to vibrate at independent frequencies.

Vocal folds are thought to decouple due to large airflow, incomplete closure of the glottis (the laryngeal opening), and/or F0 coinciding with a formant [32, 69]. Interestingly, the mean F0 of the female rejection calls in the dataset that contained biphonation was 846.8 Hz and the first formant in female snarls (likely to be the first vocal tract resonance) occurred at 824 Hz. Accordingly, biphonation in female koala rejection calls may be driven at least in part by F0 and F1 coinciding [34]. Another plausible explanation is that additional vibrating structures are responsible for producing G0. These could include the arytenoid cartilages, the ventricular folds, and the epiglottis. Though at present I can only speculate as to how female koalas produce these source features, there is good reason to assume that the ability to produce biphonation is important in the koala’s vocal communication system. For example, biphonation could make rejection calls harder to ignore by increasing the acoustic unpredictability [35, 40]. This may in turn increase the likelihood that male koalas respond to the calls, thereby inciting male-male competition during the breeding season. The relative prevalence of NLP in tonal female rejection calls might also signal the arousal state of the caller [70, 71]. These intriguing possibilities certainly merit additional exploration.

### The information content of female koala vocalisations

Female koala vocalisations varied in their individual distinctiveness. Female bellows were the most highly individualised, followed by tonal rejection calls. Snarls could not be classified using the more conservative leave-one-out procedure, and must therefore be considered as containing very few identity cues. This is surprising because snarls appear to have clear formants, and the formant pattern of mammalian vocalisations is typically highly individualised [10–13, 72]. In line with this, the formant frequency values and spacing were found to be the most individually distinctive components of female bellows. Snarls may be poorly classified according to the identity of the caller due to the lack of F0 related features and an inconsistent level of lip rounding across a given individual’s calls, which would vary the amount of vocal tract elongation and shift the entire formant pattern. The results clearly indicate that female koalas could be distinguished based on their bellows and tonal rejection calls though, and this may have adaptive significance for male koalas during the breeding season. Prior familiarity between captive male and female koalas has been shown to increase the chances of successful copulation occurring [56]. Consequently, male koalas could use identity cues present in female calls to approach potential mating partners that they are more familiar with, and with which they are most likely to copulate.

The only acoustic feature of female bellows that varied consistently according the caller’s age was minimum F0, which was higher for older females. Since F0 features of female bellows are most likely to be produced by this species’ velar vocal folds [60] I suggest that these structures loose mass and elasticity with age (as in human vocal folds [73]), and that this reduces their capacity for self-sustained periodic oscillation at lower frequencies. In contrast, and perhaps reflecting the likely different laryngeal mode of production (as opposed to the non-laryngeal velar vocal folds), the F0 features of female tonal rejection calls were all lower in older callers. This indicates that vocal fold length could be positively correlated with age in female koalas as it is in other mammals [63, 74]. These findings also accord well with work on other mammal species that has shown F0 lowers with age [19, 75]. Because the fecundity of female koalas drops with age [56] it may prove adaptive for males to use age-related variation in female calls to focus their reproductive attempts on younger females that produce higher F0 rejection calls. Playback experiments are now needed to test whether male koalas show more interest towards tonal female rejection calls with higher F0 values.

Koala bellows showed marked sex differences, with every acoustic feature that was measured differing significantly between male and female bellows. This is not surprising because source and filter-related acoustic features of human and nonhuman mammal vocalisations often vary between the sexes [11, 25, 27, 75–77]. The results of this study show that female bellows are shorter in duration, have higher mean and maximum F0, higher frequency formants and, as a consequence, higher overall formant spacing (Fig 2). Based on the findings of previous playback studies [45, 47] the relatively large sex difference in formant spacing would be perceptible to male koalas, and it would clearly be adaptive for them to discern whether a bellow comes from a potential mate (i.e. opposite sex conspecific) or a same-sex individual that is more likely to represent a competitor and potential threat. Indeed, whether a given vocalisation’s specific information content is selected for *per se*, or arises due to differences in vocal production anatomy, we would expect receivers to attend to any available information when it is adaptive for them to do so. The next step constitutes using playback experiments to determine if koalas use information on identity, age and sex in female calls when they assess whether or not to approach conspecifics in their natural environment.

## Supporting Information

S1 Datasets IBM SPSS Statistics version 20 datasets. (ZIP)
